# Improved Method of Artificial Preparation

**Published:** 1889-08

**Authors:** J. E. Thomas


*Ed. Daniel's Texas Medical Journal:*

### A METHOD OF ARTIFICIAL RESPIRATION.

*Ed. Daniel's, Texas Medical Journal:*

I have for some time been practicing a method of artificial respiration, mention of which I have never seen in print, and which I find much better than Sylvester's, or any other with which I am acquainted. It is as follows:

Have the patient in position as for Sylvester's method, with operator at patients head, and with each hand spread out on patients chest; hook the fingers under the margin of the ribs, and make firm traction upward and onward, which will produce a deep inspiration then with firm pressure downward and inward, expiration is produced. This may not be "someting new," but if it is, and you think it worthy, you may "shape it up," and publish it in your journal.

Yours, &c., J. E. Thomas.

